# ICTV Virus Taxonomy Profile: *Jingchuvirales* 2023

**DOI:** 10.1099/jgv.0.001924

**Published:** 2023-12-19

**Authors:** Jens H. Kuhn, Nolwenn M. Dheilly, Sandra Junglen, Sofia Paraskevopoulou, Mang Shi, Nicholas Di Paola

**Affiliations:** ^1^​ Integrated Research Facility at Fort Detrick, National Institute of Allergy and Infectious Diseases, National Institutes of Health, Fort Detrick, Frederick, MD 21702, USA; ^2^​ Pathogen Discovery Laboratory, Institut Pasteur, Université Paris Cité, 75015 Paris, France; ^3^​ Institute of Virology, Charité-Universitätsmedizin Berlin, Corporate Member of Freie Universität Berlin, Humboldt-Universität zu Berlin, and Berlin Institute of Health, 10117 Berlin, Germany; ^4^​ Genome Competence Center (MF1), Robert Koch Institute, 13353 Berlin, Germany; ^5^​ Sun Yat-sen University, Shenzhen 510275, PR China; ^6^​ United States Army Medical Research Institute of Infectious Diseases, Fort Detrick, Frederick, MD 21702, USA

**Keywords:** *Aliusviridae*, aqualaruvirus, charybdivirus, chu-like, *Chuviridae*, chuvirus, *Crepuscuviridae*, ICTV Report, *Jingchuvirales*, *Myriaviridae*, myriavirus, *Natareviridae*, obscuruvirus, ollusvirus, taxonomy

## Abstract

*Jingchuvirales* is an order of negative-sense RNA viruses with genomes of 9.1–15.3 kb that have been associated with arachnids, barnacles, crustaceans, insects, fish and reptiles in Africa, Asia, Australia, Europe, North America and South America. The jingchuviral genome has two to four open reading frames (ORFs) that encode a glycoprotein (GP), a nucleoprotein (NP), a large (L) protein containing an RNA-directed RNA polymerase (RdRP) domain, and/or proteins of unknown function. Viruses in the order are only known from their genome sequences. This is a summary of the International Committee on Taxonomy of Viruses (ICTV) Report on the order *Jingchuvirales* and on the families *Aliusviridae*, *Chuviridae*, *Crepuscuviridae*, *Myriaviridae* and *Natareviridae*, which are available at ictv.global/report/jingchuvirales, ictv.global/report/aliusviridae, ictv.global/report/chuviridae, ictv.global/report/crepuscuviridae, ictv.global/report/myriaviridae and ictv.global/report/natareviridae, respectively.

## Virion

Unknown.

## Genome

**Fig. 1.**
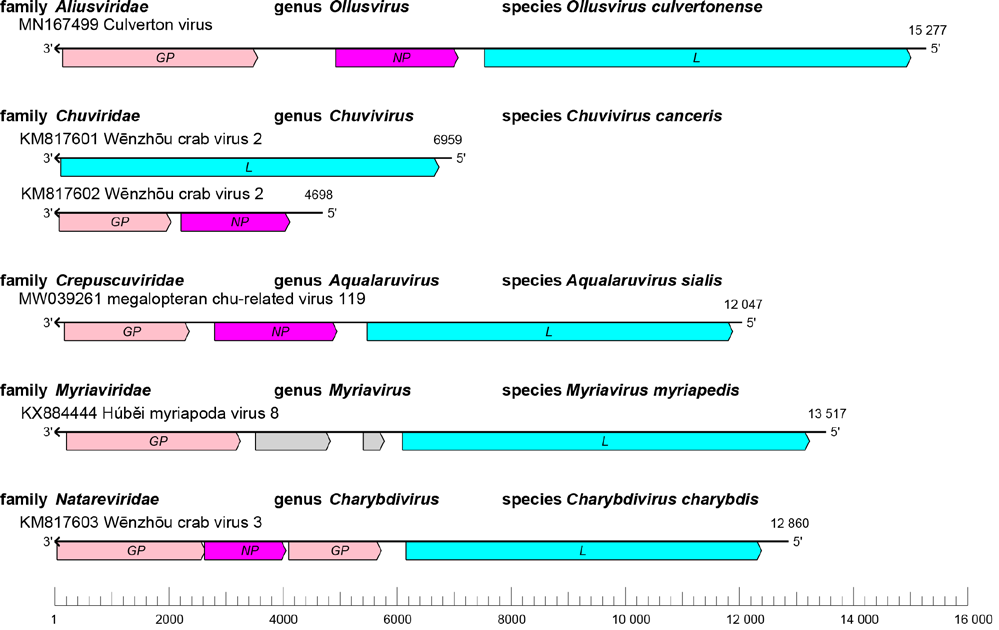
Genome organisation of representative jingchuvirals. ORFs are coloured according to the predicted protein function. *GP*, glycoprotein gene; *NP*, nucleoprotein gene; *L*, large protein gene. Grey colour indicates ORFs encoding proteins of unknown function.

The jingchuviral genome is a nonsegmented linear, nonsegmented circular, bisegmented linear, or bisegmented circular negative-sense RNA of 9.1–15.3 kb containing two to four open reading frames (ORFs) that encode a glycoprotein (GP), a nucleoprotein (NP), a large (L) protein containing an RNA-directed RNA polymerase (RdRP) domain, and/or proteins of unknown function [1–5] (Table 1, Fig. 1).

**Table 1.** Characteristics of members of the order *Jingchuvirales*

Example	Wēnzhōu crab virus 2 (S1: KM817601; S2: KM817602), species *Chuvivirus canceris*, genus *Chuvivirus*, family *Chuviridae*
Virion	Unknown
Genome	9.1–15.3 kb of nonsegmented linear, nonsegmented circular, bisegmented linear, or bisegmented circular negative-sense RNA
Replication	Unknown
Translation	Unknown
Host range	Arachnids; barnacles; crustaceans; insects; myriapods; fish; reptiles
Taxonomy	Realm *Riboviria*, kingdom *Orthornavirae*, phylum *Negarnaviricota*, class *Monjiviricetes*; the order includes >4 families, >20 genera and >57 species

## Replication

Unknown.

## Taxonomy

**Fig. 2.**
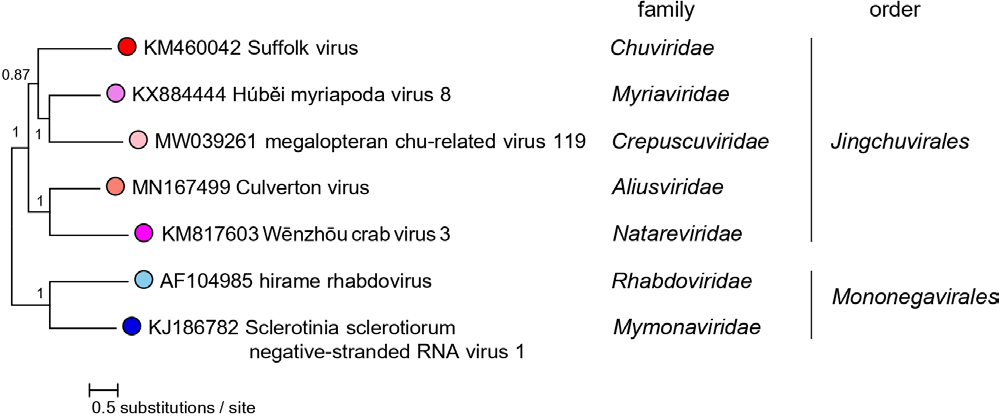
Phylogenetic relationships of jingchuvirals. Maximum-likelihood tree (midpoint-rooted) inferred using large protein gene (*L*) sequences. For details see full ICTV Report. Numbers near nodes on the trees indicate percentage bootstrap values.

Current taxonomy: ictv.global/taxonomy. The order *Jingchuvirales* includes >50 virus species in the families *Aliusviridae*, *Chuviridae*, *Crepuscuviridae*, *Myriaviridae* and *Natareviridae* for viruses that infect arachnids, barnacles, crustaceans, insects, fish and reptiles (Fig. 2).

## Resources

Full ICTV Report on the order *Jingchuvirales* and the families *Aliusviridae*, *Chuviridae*, *Crepuscuviridae*, *Myriaviridae* and *Natareviridae*: ictv.global/report/jingchuvirales, ictv.global/report/aliusviridae, ictv.global/report/chuviridae, ictv.global/report/crepuscuviridae, ictv.global/report/myriaviridae and ictv.global/report/natareviridae, respectively.
